# Quality of life predicts survival in patients with non-small cell lung cancer

**DOI:** 10.1186/1471-2458-12-790

**Published:** 2012-09-15

**Authors:** Tsai-Chung Li, Chia-Ing Li, Chun-Hua Tseng, Kuan-Shin Lin, Sing-Yu Yang, Chih-Yi Chen, Te-Chun Hsia, Yih-Dar Lee, Cheng-Chieh Lin

**Affiliations:** 1 Graduate Institute of Biostatistics, College of Public Health, China Medical University, Taichung, Taiwan; 2 Department of Healthcare Administration, College of Health Science, Asia University, Taichung, Taiwan; 3 Department of Medical Research, China Medical University Hospital, Taichung, Taiwan; 4 School of Medicine, College of Medicine, China Medical University, Taichung, Taiwan; 5 Department of Public Health, College of Public Health, China Medical University, Taichung, Taiwan; 6 Graduate Institute of Health Care Administration, College of Public Health, China Medical University, Taichung, Taiwan; 7 Cancer Center, China Medical University Hospital, Taichung, Taiwan; 8 Department of Internal Medicine, China Medical University Hospital, Taichung, Taiwan; 9 Bristol-Myers Squibb (Taiwan) Ltd, Global Development & Medical Affair, Taipei, Taiwan; 10 Department of Psychiatry, Medical College, National Cheng-Kung University, Tainan, Taiwan; 11 Department of Family Medicine, China Medical University Hospital, Taichung, Taiwan

**Keywords:** Lung cancer, HRQOL, Prognostic factors, Survival

## Abstract

**Background:**

Patients with non-small cell lung cancer (NSCLC) have a poor prognosis. The objective of this study was to examine the relationship of EORTC QLQ-C30 and QLQ-LC13 and survival in patients with NSCLC undergoing different treatments.

**Methods:**

Investigators conducted a health-related quality of life (HRQOL) survey of 488 patients with NSCLC: 162 patients undergoing surgery, 312 patients without surgery, and their survival status was prospectively followed up. EORTC QLQ-C30 and QLQ-LC13 scores and clinical variables at baseline were analyzed using Cox’s proportional hazard regression to identify factors that influenced survival.

**Results:**

Median survival of these 474 patients was 9.82 months. After adjustment, emotional functioning scale, and symptom scales of pain and nausea/vomiting are associated with survival in NSCLC patients with surgery whereas social functioning scale, and symptom scales for fatigue, appetite loss, and financial problems had a significant impact on survival in NSCLC patients without surgery. The results of multivariate analysis showed that none of QLQ-LC13 scales are significant predictors of survival. After simultaneously considering these scales, we found significant independent predictors of survival were nausea/vomiting (HR = 0.11, 95% CI = 0.02-0.63 for score >0 compared with =0) in NSCLC patients with surgery and appetite loss (HR = 1.77, 95% CI = 1.26-2.49 for score >0 compared with =0) in NSCLC patients without surgery.

**Conclusions:**

HRQOL provides additional predictive information that supplements traditional clinical factors, and is a new prognostic indicator for survival of NSCLC patients under different treatments.

## Background

Lung cancer (LC) is the leading cause of cancer-associated mortality worldwide. In 2011, malignant neoplasm was the number one leading cause of death in Taiwan, accounting for more than 20% of total deaths [1]. Lung cancer was ranked as the second most common cancer in men, accounting for about 21.2% of all cancer deaths, and was the most common cancer in women, accounting for about 16.7% of all cancer deaths.

A growing body of research shows that self-perceptions of health are linked to mortality, even when more objective health measures, such as morbidity [2,3], social support [3], and health behaviors [4] are controlled. Health-related quality of life (HRQOL) and its assessment have become increasingly important in the field of oncology. HRQOL is an important aspect of cancer care. It has been acknowledged as an important end point in cancer clinical trials and clinical practice, along with the traditional end points, including tumor response rate, disease-free survival, and overall survival. Several studies have investigated the prognostic factors of patients with lung cancer, and these have been reported to enable the differentiation of patients with favorable and adverse prognoses [5-8]; analyses of advanced non-small cell lung carcinoma treated with chemotherapy [5] or with radiotherapy [6], of new cases of all cancer types and treatments [7], and of advanced lung carcinoma of all cancer types with chemotherapy [8] have been undertaken.

There is an increasing need for the Chinese version HRQOL instruments for cancer patients due to increasing incidence rates of cancer in Taiwan. Although the Chinese version of the European Organization for Research and Treatment of Cancer (EORTC) QLQ-C30 and lung cancer-specific (LC-13) scales were available, their relationships with survival in lung cancer patients have never been reported. Therefore, the objective of this study was to examine the relationship of the EORTC QLQ-C30 and LC-13 scales and survival in Taiwanese patients with stages I-IV non-small cell lung cancer (NSCLC).

## Methods

### NSCLC patients

From July 2004 to December 2007, we consecutively contacted NSCLC patients undergoing active chemotherapy, surgery or post-therapy follow-up in an inpatient setting or at the outpatient clinic of the Department of Oncology at China Medical University Hospital and Taichung Veterans General Hospital, two medical centers in central Taiwan. NSCLC patients in this cross-sectional study were initially recruited to participate in a known-groups validation study of the EORTC QLQ-C30 and QLQ-LC13. The eligibility criteria for inclusion were a clinical diagnosis of NSCLC with local, advanced, or metastatic lung cancer; American Joint Committee on Cancer/Union International Cancer Center (AJCC/UICC) stages I-IV; the ability to read, write and speak Mandarin or Taiwanese; informed consent; and no major disabling medical or psychiatric conditions that would substantially impair cognitive functioning. Patients who declined to participate were excluded. Patients were instructed to complete the questionnaires themselves. Patients who had difficulty in completing the questionnaires were assisted by interviewers fluent in both Mandarin and Taiwanese. This study was approved by the Human Research Committee of China Medical University Hospital. Written informed consent was obtained from each participant.

### Prognostic clinical variables

We abstracted data on tumor characteristics, such as date of diagnosis, histology, tumor type, stage, and treatment, and patient characteristics like gender, date of birth and co-morbidity at the time of diagnosis. We only included those cases with histology being categorized as NSCLC. Tumor type was categorized as squamous cell carcinoma, adenocarcinoma, and undifferentiated carcinoma. Stage of lung cancer was defined by location and extent of primary and metastatic tumor involvement. The standard AJCC/UICC system was used for staging [9]. Stage of lung cancer was differentiated as I or II, III (a or b), or IV, with the higher stage representing more advanced disease. Treatment status was classified as surgery, chemotherapy or post-therapy follow-up. Usually, NSCLC patients who did not have completely resected stage IA and IB would receive adjuvant therapy and NSCLC patients who are not candidates for surgery or who refuse surgery, curative intent chemotherapy or radiotherapy is used. Cancer duration was derived by calculating the difference between the date of entry and date of diagnosis, using month as the unit.

### Instruments

The EORTC QLQ-C30 [10] and QLQ-LC13 [11] were used. The EORTC QLQ-C30 is a core cancer-specific questionnaire containing 30 items on patients’ functioning, global quality of life (QOL), and disease- and treatment-related symptoms. The QLQ-LC13 is a site-specific questionnaire consisting of 13 items on lung cancer symptoms (cough, haemoptysis, dyspnoea, site-specific pain) and its treatment-related side-effects (sore mouth, dysphagia, peripheral neuropathy, alopecia). Previous study showed that overall validation results for the Chinese version of the EORTC QLQ-C30 and QLQ-LC13 confirmed it as a reliable and valid questionnaire for assessing lung cancer-specific HRQOL in Taiwan [12]. Patients receiving chemotherapy completed questionnaires between the first cycle of therapy and the end of the 6^th^ cycle. Patients undergoing surgery completed the surveys between one and 4 weeks after surgery. Patients free of recurrent disease completed the questionnaires at a follow-up visit.

### Main outcome measure

The primary outcome measure was survival due to any cause in the months following baseline assessment of HRQOL. Survival was ascertained by patients’ family members through telephone follow-up. For non-respondents, vital status was assessed by linking to the National Mortality database through December 31, 2007 using gender, identification number, and date of birth. For individuals reported deceased by family members, the date of death and cause of death were confirmed using the same process. The precise date of death along with the date of entry was used to calculate the event time. Those who did not die were defined as censored and their data were censored on December 31, 2007.

### Statistical analysis

The Cox proportional hazards model was used to assess the relative risk of survival in terms of the 2 HRQOL instruments. First, we categorized the scales of the QLQ C-30 and LC-13 according to their tertiles, and dichotomized the single-item symptom scales. Then, we evaluated the crude risk of survival separately for each scale, and added age, gender, stages of cancer, treatment type, duration of cancer, and lung cancer subtype. Next, we used the continuous variables of the QLQ C-30 and LC-13 scales to test linear trends. Finally, five models were examined. In the first model, the relative hazards of survival of all clinical variables were examined simultaneously. In the second model, the functioning scales of the QLQ C-30 were examined simultaneously by adjusting for clinical parameters in the first model. In the third model, the relative hazards of survival were estimated simultaneously for symptom scales of QLQ C-30 while considering all clinical parameters. In the fourth model, the relative hazards of survival of the symptom scales of the QLQ LC-13 were examined simultaneously by controlling for clinical parameters. In the last model, the significant scales of the QLQ C-30 and LC-13 in 2–4 models were examined simultaneously, while including the relative hazards of survival with the clinical parameters in the model. Kaplan-Meier estimates were used to obtain the proportion of patients who had an event during follow-up for those significant scales of QLQ-C30 and LC-13 in model 5 and log-rank tests were used to compare whether the survival functions across subgroups were different. All analyses were conducted using SAS, version 9.1 (SAS Institute Inc. Cary NC).

## Results

### Patient population

Four hundred seventy-eight NSCLC patients (162 receiving surgery and 312 not receiving surgery) participated in this study. The socio-demographic and clinical data and their relationship to survival stratified by surgery status are presented in Table 1. Age, clinical stage, primary tumor, regional lymph nodes, distant metastasis, and type of treatment were significantly associated with survival in both groups. In addition, female was significantly associated with longer survival (HR = 0.63, 95% CI = 0.45-0.88) and cancer duration <1 year was associated with worse survival (2.30, 1.43-3.70 for new cases; 2.36, 1.49-3.75 for <1 year) in patients without surgery.

**Table 1:** Baseline characteristics of NSCLC patients stratified by surgery status and their relation to survival time

**Variables**	**Surgery (N = 162)**									**Non-surgery (N = 312)**
	**No. (%)**					**No. of deaths (%)**	**HR (95% C.I.)**	**P value**	**No. (%)**	**No. of deaths (%)**	**HR (95% C.I.)**	**P value**
Gender												
Male	102 (62.96)					26 (25.49)	1.00		206 (66.03)	118 (57.28)	1.00	
Female	60 (37.04)					9 (15.00)	0.73 (0.34-1.55)	0.41	106 (33.97)	50 (47.17)	0.63 (0.45-0.88)	0.006
Age (yrs)												
<50	32 (19.75)					3 (9.38)	1.00		48 (15.39)	22 (45.38)	1.00	
50 ≤ Age < 60	32 (19.75)					6 (18.75)	1.42 (0.36-5.70	0.62	65 (20.83)	34 (52.31)	1.38 (0.81-2.36)	0.24
60 ≤ Age < 70	45 (27.78)					12 (26.67)	2.01 (0.57-7.13)	0.28	91 (29.17)	47 (51.65)	1.24 (0.75-2.05)	0.41
70 ≤ Age < 80	50 (30.86)					11 (22.00)	1.90 (0.53-6.82)	0.33	90 (28.85)	52 (57.78)	1.44 (0.87-2.37)	0.15
≥80	3 (1.85)					3 (100.00)	8.38 (1.69-41.66)	0.009	18 (5.77)	13 (72.22)	2.80 (1.41-5.57)	0.003
Cancer Duration (yrs)												
New cases	102 (62.96)					17 (16.67)	1.42 (0.52-3.89)	0.49	135 (43.27)	66 (48.89)	2.30 (1.43-3.70)	<0.001
Cancer duration <1	41 (25.31)					13 (31.71)	1.78 (0.63-5.00)	0.27	124 (39.74)	78 (62.90)	2.36 (1.49-3.75)	<0.001
Cancer duration ≥1	19 (11.73)					5 (26.32)	1.00		53 (16.99)	24 (45.28)	1.00	
Type of Cancer												
Adenocarcinoma	100 (61.73)					19 (19.00)	1.00		195 (62.50)	98 (50.26)	1.00	
Squamous Cell	51 (31.48)					13 (25.49)	1.17 (0.58-2.38)	0.66	92 (29.49)	55 (59.78)	1.20 (0.86-1.67)	0.28
Other	11 (6.79)					3 (27.27)	3.57 (1.05-12.16)	0.04	25 (8.01)	15 (60.00)	1.14 (0.66-1.96)	0.65
Clinical Stage												
IA, IB, IIA, IIB	115 (70.99)					18 (15.65)	1.00		42 (13.46)	12 (28.57)	1.00	
IIIA, IIIB	39 (24.07)					12 (30.77)	2.36 (1.13-4.90)	0.02	97 (31.09)	54 (55.67)	4.83 (2.57-9.08)	<0.001
IV	8 (4.94)					5 (62.50)	7.44 (2.74-20.20)	<0.001	173 (55.45)	102 (58.96)	6.08 (3.30-11.21)	<0.001
Primary Tumor^a^												
T1	35 (21.74)					5 (14.29)	1.00		23 (7.54)	12 (52.17)	1.00	
T2	88 (54.66)					17 (19.32)	1.44 (0.53-3.90)	0.48	81 (26.56)	32 (39.51)	0.83 (0.43-1.61)	0.58
T3	20 (12.42)					7 (35.00)	2.18 (0.69-6.88)	0.18	28 (9.18)	15 (53.57)	1.30 (0.61-2.78)	0.50
T4	18 (11.18)					6 (33.33)	3.21 (0.97-10.58)	0.06	173 (56.72)	105 (60.69)	2.10 (1.15-3.83)	0.02
Regional Lymph Nodes^a^												
N0	104 (67.10)					16 (15.38)	1.00		67 (23.26)	28 (41.79)	1.00	
N1	29 (18.71)					8 (27.59)	1.80(0.77-4.22)	0.17	18 (6.25)	7 (38.89)	1.00 (0.44-2.30)	0.99
N2	20 (12.90)					6 (30.00)	3.10(1.21-7.96)	0.02	79 (27.43)	45 (56.96)	2.49 (1.54-4.02)	<0.001
N3	2 (1.29)					1 (50.00)	1.60(0.21-12.1)	0.65	124 (43.06)	80 (64.52)	3.69 (2.36-5.76)	<0.001
Distant Metastasis												
M0	152 (93.83)					29 (19.08)	1.00		139 (44.55)	65 (46.76)	1.00	
M1	10 (6.17)					6 (60.00)	3.29 (1.36-7.93)	0.008	173 (55.45)	103 (59.54)	2.12 (1.54-2.92)	<0.001
Type of Treatment												
Pneumonectomy												
No	132 (90.41)					24 (18.18)	1.00					
Yes	14 (9.59)					1 (7.14)	0.17 (0.02-1.29)	0.09				
Lobectomy/Bilobectomy												
No	59 (40.41)					14 (23.73)	1.00					
Yes	87 (59.59)					11 (12.64)	0.81 (0.37-1.79)	0.60				
Wedge/Segmental resection												
No	98 (67.12)					11 (11.22)	1.00					
Yes	48 (32.88)					14 (29.17)	2.23 (1.01-4.92)	0.05				
Adjuvant Therapy												
No	61 (53.04)					10 (16.39)	1.00					
Yes	54 (46.96)					8 (14.81)	1.32 (0.52-3.35)	0.56				
Chemotherapeutic agents^a^												
Iressa									25 (9.80)	12 (48.00)	1.00	
Navelbine									129 (50.59)	85 (65.89)	1.69 (0.63-4.49)	0.30
Gemzar									2 (0.78)	2 (100.00)	2.57 (1.12-5.88)	0.03
Taxol									68 (26.67)	37 (54.41)	3.48 (0.7-17.29)	0.13
Taxotere									20 (7.84)	6 (30.00)	2.45 (1.03-5.81)	0.04
Other									11 (4.31)	7 (63.64)	3.88 (1.3-11.59)	0.01
Radiotherapy												
No									264 (87.42)	138 (52.27)	1.00	
Yes									38 (12.58)	22 (57.89)	1.84 (1.16-2.90)	0.009

^a^: sample size was not equal to 162 due to missing data; *CI*, confidence interval; *NSCLC*, non-small cell lung cancer.

### Health-related quality of life and survival

Table 2 shows the results of univariate and multivariate analyses that examined whether the various QOL-C30 scores were significant for overall survival stratified by surgery status. After multivariate adjustment for adjusting for age, gender, cancer duration, type of cancer and cancer clinical stage of TNM, emotional functioning scale, and symptom scales of pain and nausea/vomiting are associated with survival in NSCLC patients with surgery whereas social functioning scale, and symptom scales for fatigue, appetite loss, and financial problems had a significant impact on survival in NSCLC patients without surgery. And there existed a significant linear trend for fatigue scale. For scales of the QLQ-LC13, the results of univariate analysis showed that the sore mouth in patients with surgery, and hemoptysis, and dysphagia scales of the QLQ-LC13 in NSCLC patients without surgery had a significant impact on survival Table 3. The results of multivariate analysis showed that none of these scales remained statistically significant after adjustment.

**Table 2:** Univariate and multivariate Cox’s analyses of baseline EORTC QLQ-C30 for overall survival stratified by surgery status

**Variables**	**Surgery (N = 162)**				**Non-surgery (N = 312)**			
	**N**	**# of death**	**Crude HR (95%C.I.)**	**Adjusted HR (95%C.I.)**	**N**	**# of death**	**Crude (95%C.I.)**	**Adjusted HR (95%C.I.)**
***QLQ-C30***								
Physical Functioning (<100 as reference)	81	25	1.00	1.00	225	130	1.00	1.00
=100	81	10	0.52 (0.25-1.09)	0.61 (0.22-1.74)	84	35	0.67 (0.46-0.98)*	0.89 (0.59-1.35)
Role Functioning (<100 as reference)	27	5	1.00	1.00	105	61	1.00	1.00
=100	135	30	1.30 (0.51-3.36)	2.73 (0.66-11.39)	205	105	0.71 (0.52-0.98)*	1.08 (0.75-1.54)
Emotional Functioning (<83.33 as reference)	62	12	1.00	1.00	122	63	1.00	1.00
83.33≤EFS < 100	44	11	1.29 (0.57-2.92)	3.36 (1.05-10.69)*	85	53	1.15 (0.8-1.66)	1.20 (0.81-1.78)
=100	56	12	1.06 (0.48-2.36)	2.00 (0.67-5.96)	105	52	0.96 (0.66-1.39)	1.11 (0.75-1.66)
Cognitive Functioning (<100 as reference)	84	19	1.00	1.00	163	90	1.00	1.00
=100	78	16	0.82 (0.42-1.59)	1.21 (0.53-2.76)	149	78	1.00 (0.74-1.35)	1.11 (0.79-1.56)
Social Functioning (<100 as reference)	65	16	1.00	1.00	159	94	1.00	1.00
=100	96	19	0.76 (0.39-1.48)	1.41 (0.57-3.53)	151	73	0.57 (0.42-0.78)***	0.70 (0.50-0.99)*
Global QOL (≤50 as reference)	54	16	1.00	1.00	166	98	1.00	1.00
50 < QLS≤66.67	55	7	0.43 (0.18-1.05)	0.51 (0.17-1.53)	72	40	0.75 (0.52-1.08)	0.90 (0.61-1.32)
>66.67	52	11	0.72 (0.33-1.55)	0.98 (0.34-2.85)	73	29	0.45 (0.29-0.68)***	0.79 (0.50-1.26)
Fatigue (≤11.12 as reference)	96	15	1.00	1.00	99	46	1.00	1.00
11.12 < FAS≤33.34	36	10	1.65 (0.74-3.67)	1.78 (0.71-4.48)	111	52	1.37 (0.92-2.04)	1.49 (0.98-2.27)
>33.34	30	10	1.83 (0.82-4.06)	1.07 (0.37-3.09)	102	70	2.52 (1.73-3.68)***	2.00 (1.34-3.00)***
Pain (=0 as reference)	89	19	1.00	1.00	121	68	1.00	1.00
0 < Pain≤22.22	36	5	0.48 (0.18-1.28)	0.20 (0.05-0.78)*	73	34	0.73 (0.49-1.11)	0.82 (0.53-1.29)
>22.22	37	11	0.98 (0.47-2.07)	0.53 (0.19-1.48)	118	66	1.18 (0.84-1.66)	1.02 (0.70-1.47)
Nausea/Vomiting (=0 as reference)	147	31	1.00	1.00	248	124	1.00	1.00
>0	15	4	0.79 (0.28-2.24)	0.10 (0.02-0.56)**	63	43	1.44 (1.02-2.04)*	0.98 (0.66-1.44)
Dyspnoea (=0 as reference)	114	17	1.00	1.00	156	72	1.00	1.00
>0	48	18	1.80 (0.93-3.51)	1.69 (0.70-4.07)	155	96	1.49 (1.10-2.02)*	1.08 (0.77-1.51)
Insomnia (=0 as reference)	93	18	1.00	1.00	154	80	1.00	1.00
>0	68	17	1.29 (0.67-2.51)	1.62 (0.72-3.64)	152	82	0.93 (0.69-1.27)	0.99 (0.71-1.37)
Appetite Loss (=0 as reference)	125	28	1.00	1.00	188	84	1.00	1.00
>0	37	7	0.71 (0.31-1.63)	0.39 (0.13-1.20)	124	84	2.26 (1.66-3.07)***	1.79 (1.27-2.52)***
Constipation (=0 as reference)	123	23	1.00	1.00	209	103	1.00	1.00
>0	39	12	1.86 (0.92-3.75)	1.41 (0.51-3.92)	102	65	1.38 (1.01-1.88)*	1.12 (0.80-1.57)
Diarrhoea (=0 as reference)	147	31	1.00	1.00	263	136	1.00	1.00
>0	15	4	1.12 (0.40-3.18)	0.49 (0.07-3.27)	48	32	1.25 (0.85-1.84)	1.28 (0.85-1.95)
Financial Difficulties (=0 as reference)	119	23	1.00	1.00	216	103	1.00	1.00
>0	41	11	1.52 (0.74-3.12)	0.84 (0.28-2.55)	94	64	1.83 (1.34-2.51)***	1.55 (1.08-2.20)*

Adjusted for age, sex, cancer duration, type of cancer and clinical stage (TNM stage).

*:P < 0.05, **:P < 0.01, ***:P < 0.001.

**Table 3:** Univariate and multivariate Cox’s analyses of baseline EORTC QLQ-LC13 for overall survival stratified by surgery status

**Variables**	**Surgery (N = 162)**				**Non-surgery (N = 312)**			
	**N**	**# of death**	**Crude HR (95%C.I.)**	**Adjusted HR (95%C.I.)**	**N**	**# of death**	**Crude HR (95%C.I.)**	**Adjusted HR (95%C.I.)**
***QLQ-LC13***								
Dyspnoea (=0 as reference)	82	8	1.00	1.00	107	44	1.00	1.00
>0	79	26	2.03 (0.91-4.51)	1.46 (0.51-4.16)	201	120	1.32 (0.93-1.86)	1.16 (0.80-1.69)
Cough (=0 as reference)	52	10	1.00	1.00	82	43	1.00	1.00
>0	109	24	1.31 (0.63-2.75)	0.80 (0.33-1.91)	230	125	1.19 (0.84-1.69)	0.97 (0.68-1.40)
Hemoptysis (=0 as reference)	146	30	1.00	1.00	261	142	1.00	1.00
>0	15	4	2.23 (0.78-6.37)	3.01 (0.81-11.18)	50	26	1.57 (1.03-2.39)*	1.04 (0.65-1.69)
Sore Mouth (=0 as reference)	154	30	1.00	1.00	278	147	1.00	1.00
>0	7	4	3.59 (1.26-10.20)*	0.87 (0.14-5.48)	33	21	1.16 (0.73-1.84)	1.03 (0.60-1.76)
Dysphagia (=0 as reference)	144	29	1.00	1.00	257	135	1.00	1.00
>0	17	5	1.31 (0.51-3.40)	0.58 (0.15-2.27)	53	32	1.73 (1.18-2.55)**	1.06 (0.68-1.64)
Peripheral Neuropathy (=0 as reference)	108	23	1.00	1.00	213	109	1.00	1.00
>0	53	11	0.86 (0.42-1.77)	1.03 (0.41-2.59)	97	59	1.10 (0.80-1.51)	1.03 (0.73-1.46)
Hair Loss (=0 as reference)	140	29	1.00	1.00	262	137	1.00	1.00
>0	21	5	1.01 (0.39-2.62)	0.32 (0.07-1.44)	47	29	0.99 (0.66-1.47)	1.11 (0.71-1.73)
Chest Pain (=0 as reference)	107	20	1.00	1.00	183	94	1.00	1.00
>0	54	14	1.05 (0.53-2.09)	0.52 (0.21-1.32)	128	73	1.07 (0.79-1.45)	0.97 (0.70-1.34)
Pain in Arm or Shoulder (=0 as reference)	123	25	1.00	1.00	217	114	1.00	1.00
>0	37	9	0.86 (0.40-1.84)	1.19 (0.45-3.14)	93	53	1.20 (0.87-1.67)	0.96 (0.66-1.39)
Other Pain Sites (=0 as reference)	117	26		1.00	193	106	1.00	1.00
>0	41	7	0.60 (0.26-1.39)	0.38 (0.12-1.19)	106	53	1.02 (0.74-1.42)	0.96 (0.67-1.38)

Adjusted for age, sex, cancer duration, type of cancer and clinical stage (TNM stage).

*:P < 0.05, **:P < 0.01, ***:P < 0.001.

### Hierarchical Cox’s proportional hazard models

Table 4 shows the hierarchical Cox’s proportional hazard models in NSCLC patients with surgery. The results for model 1 indicated that age, type of cancer, and regional lymph nodes were significant prognostic factors for survival, and model 2 showed that emotional functioning scale of the QLQ C-30 was a strong predictor of subsequent survival, after adjusting for all other variables in the model (3.36, 1.05-10.69 for a score 83.33-100 compared to ≤83.33). The results for model 3 showed that the effect of the QLQ C-30 scales for nausea/vomiting (0.12, 0.02-0.71 for score >0 compared with score of 0) was statistically significant. Because none of adjusted HR for QLQ-LC13 scale was statistically significant, we fit model 5 directly. As model 5 shows, when significant scales of the QLQ C-30 were considered simultaneously, nausea/vomiting was the only scale with a significant effect on survival (0.11, 0.02-0.63 for a score >0 compared to a score of 0) after controlling for all other variables in the model.

**Table 4:** Evaluation of the independent contribution of retained baseline variables for each prognostic score in NSCLC patients with surgery

**Variables**	**Surgery HR (95%C.I.)**			
	**Model 1**	**Model 2**	**Model 3**	**Model 4**
Gender (=Male as reference)				
Female	0.90 (0.31-2.66)	1.27 (0.41-3.89)	1.62 (0.56-4.68)	1.86 (0.63-5.56)
Age (yrs) (<50 as reference)				
50 ≤ Age < 60	1.54 (0.27-8.91)	1.51 (0.26-8.93)	2.24 (0.37-13.52)	2.55 (0.43-15.11)
60 ≤ Age < 70	3.71 (0.65-21.23)	3.01 (0.48-19.06)	3.88 (0.73-20.71)	3.50 (0.60-20.30)
70 ≤ Age < 80	3.32 (0.57-19.31)	2.84 (0.46-17.65)	3.53 (0.59-20.98)	3.68 (0.63-21.63)
≥80	41.03 (4.84-347.54)***	55.83 (5.76-541.35)***	168.87 (13.26-2150.64)***	283.27 (21.01-3819.27)***
Cancer duration (yrs) (≥1 as reference)				
New case	2.12 (0.65-6.92)	2.13 (0.64-7.03)	2.67 (0.70-10.12)	3.34 (0.88-12.63)
Cancer duration <1	2.24 (0.60-8.40)	2.16 (0.55-8.49)	3.71 (0.84-16.41)	4.78 (1.05-21.74)*
Type of Cancer (Adenocarcinoma as reference)				
Squamous Cell	1.76 (0.66-4.68)	2.39 (0.86-6.7)	2.57 (0.94-7.02)	3.62 (1.21-10.86)*
Other	5.09 (1.03-25.12)*	6.01 (1.00-35.92)*	8.25 (1.58-43.09)*	6.64 (1.12-39.39)*
Primary Tumor (T1 as reference)				
T2	0.84 (0.28-2.50)	1.02 (0.33-3.20)	1.33 (0.43-4.17)	1.47 (0.45-4.81)
T3	0.94 (0.23-3.78)	1.17 (0.29-4.80)	1.38 (0.35-5.48)	1.63 (0.40-6.64)
T4	1.54 (0.35-6.79)	2.55 (0.52-12.56)	4.42 (0.92-21.22)	5.40 (0.99-29.51)
Regional Lymph Nodes (N0 as reference)				
N1	2.34 (0.85-6.45)	2.98 (1.02-8.68)*	2.73 (1.01-7.42)*	3.80 (1.34-10.80)*
N2	5.32 (1.70-16.64)**	6.17 (1.88-20.19)**	5.65 (1.68-18.96)**	7.39 (2.15-25.43)**
N3	6.74 (0.51-89.64)	16.60 (0.98-282.16)	4.55 (0.32-65.68)	9.75 (0.62-153.58)
Distant Metastasis (M0 as reference)				
M1	1.73 (0.27-11)	1.07 (0.16-7.39)	1.51 (0.23-9.80)	1.79 (0.29-11.21)
***QLQ-C30***				
Emotional Functioning (<83.33 as reference)				
83.33≤EFS < 100		3.36 (1.05-10.69)*		2.56 (0.82-7.96)
=100		2.00 (0.67-5.96)		1.17 (0.39-3.56)
Pain (=0 as reference)				
0 < Pain≤22.22			0.24 (0.06-1.01)	
>22.22			0.83 (0.30-2.25)	
Nausea/Vomiting (=0 as reference)				
>0			0.12 (0.02-0.71)*	0.11 (0.02-0.63)*
−2 log likelihood	242.88	238.26	228.77	230.68

*:P < 0.05, **:P < 0.01, ***:P < 0.001.

Table 5 shows the hierarchical Cox’s proportional hazard models in NSCLC patients without surgery. The results for model 1 indicated that gender, age, cancer duration, type of cancer, regional lymph nodes, and distant metastasis were significant prognostic factors for survival, and model 2 showed that social functioning scale of the QLQ C-30 was a strong predictor of subsequent survival, after adjusting for all other variables in the model (0.70, 0.50-0.99 for a score 100 compared to <100). The results for model 3 showed that the effect of the QLQ C-30 scales for appetite loss (1.62, 1.12-2.36 for score >0 compared with score of 0) were significant. Because none of adjusted HR for QLQ-LC13 scale was statistically significant, we fit model 5 directly. As model 5 shows, when significant scales of the QLQ C-30 were considered simultaneously, the effects of appetite loss on survival remained significant (1.77, 1.26-2.49) after controlling for all other variables in the model.

**Table 5:** Evaluation of the independent contribution of retained baseline variables for each prognostic score in NSCLC patients without surgery

**Variables**	**Non-surgery HR (95%C.I.)**			
	**Model 1**	**Model 2**	**Model 3**	**Model 4**
Gender (=Male as reference)				
Female	0.53 (0.36-0.79)**	0.57 (0.38-0.85)**	0.52 (0.35-0.78)**	0.55 (0.37-0.82)**
Age (yrs) (<50 as reference)				
50 ≤ Age < 60	1.92 (1.08-3.39)*	2.02 (1.15-3.56)*	2.46 (1.38-4.36)**	2.31 (1.3-4.11)**
60 ≤ Age < 70	1.45 (0.86-2.44)	1.47 (0.88-2.47)	1.40 (0.82-2.39)	1.41 (0.84-2.37)
70 ≤ Age < 80	1.80 (1.07-3.03)*	1.91 (1.13-3.24)*	1.93 (1.12-3.32)*	1.94 (1.15-3.29)*
≥80	3.70 (1.73-7.89)***	4.53 (2.10-9.75)***	4.65 (2.14-10.08)***	4.73 (2.19-10.24)***
Cancer duration (yrs) (≥1 as reference)				
New case	1.19 (0.69-2.05)	1.05 (0.61-1.82)	1.28 (0.74-2.22)	1.16 (0.66-2.01)
Cancer duration <1	1.79 (1.08-2.97)*	1.70 (1.02-2.82)*	1.62 (0.97-2.69)	1.62 (0.97-2.69)
Type of Cancer (Adenocarcinoma as reference)				
Squamous Cell	0.91 (0.62-1.34)	0.93 (0.62-1.37)	0.87 (0.59-1.30)	0.92 (0.62-1.37)
Other	1.20 (0.65-2.20)	1.42 (0.75-2.68)	1.23 (0.63-2.38)	1.23 (0.64-2.36)
Primary Tumor (T1 as reference)				
T2	0.57 (0.28-1.16)	0.60 (0.3-1.21)	0.54 (0.27-1.08)	0.54 (0.27-1.1)
T3	1.20 (0.55-2.64)	1.20 (0.54-2.65)	1.19 (0.54-2.64)	1.17 (0.53-2.58)
T4	1.28 (0.67-2.46)	1.32 (0.68-2.55)	1.15 (0.59-2.23)	1.22 (0.63-2.35)
Regional Lymph Nodes (N0 as reference)				
N1	1.06 (0.45-2.49)	0.99 (0.42-2.31)	1.12 (0.47-2.66)	1.01 (0.43-2.37)
N2	2.12 (1.24-3.61)**	1.99 (1.17-3.38)*	1.82 (1.05-3.14)*	1.87 (1.09-3.20)*
N3	2.29 (1.34-3.92)**	2.14 (1.26-3.65)**	1.85 (1.06-3.22)*	1.84 (1.06-3.18)*
Distant Metastasis (M0 as reference)				
M1	1.54 (1.04-2.28)*	1.52 (1.03-2.25)*	1.37 (0.91-2.07)	1.47 (0.99-2.19)
***QLQ-C30***				
Social Functioning (<100 as reference)				
=100		0.70 (0.50-0.99)*		0.73 (0.52-1.03)
Fatigue (≤11.12 as reference)				
11.12 < FAS≤33.34			1.19 (0.77-1.86)	
>33.34			1.45 (0.92-2.28)	
Appetite Loss (=0 as reference)				
>0			1.62 (1.12-2.36)*	1.77 (1.26-2.49)**
Financial Difficulties (=0 as reference)				
>0			1.43 (0.99-2.06)	
−2 log likelihood	1508.53	1490.90	1475.39	1480.24

*:P < 0.05, **:P < 0.01, ***:P < 0.001.

### Survival curves

**Figure 1:**
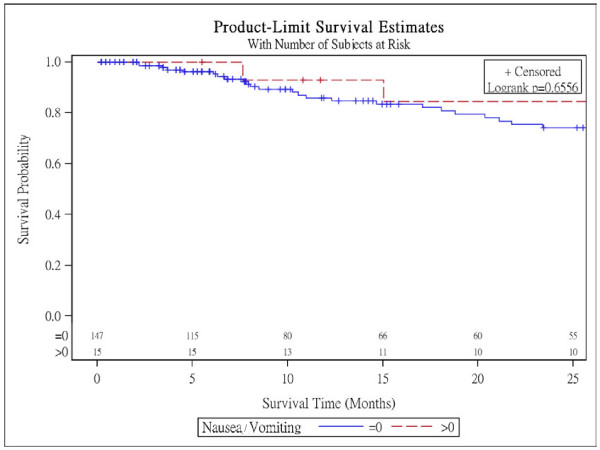
Survival curves for the baseline nausea/vomiting scores in NSCLC patients with surgery.

**Figure 2:**
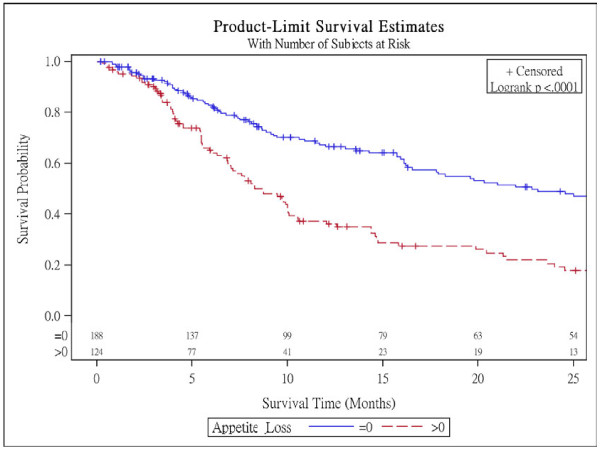
Survival curves for the baseline appetite loss scores in NSCLC patients without surgery.

Figure 1 presents Kaplan-Meier survival curves within subgroups defined by nausea/vomiting scales in NSCLC patients with surgery and log-rank tests reveal that there are no difference within subgroups for these 3 scales. Figure 2 shows Kaplan-Meier survival curves within subgroups defined by appetite loss and it is significantly different (p < 0.001).

## Discussion

The present study was the first one examining the relationship between the Chinese version of the EORTC QLQ-C30 and QLQ-LC13 and all-cause survival while adjusting for an array of clinical parameters in a sample of NSCLC patients stratified by surgery status. We demonstrated that HRQOL measured by the QLQ C-30 was a strong and independent predictor of overall survival time. When we considered the functioning and symptom scales of the QLQ C-30 and QLQ LC-13 separately, the emotional functioning scale and symptom scales of pain, nausea/vomiting were strong predictors with regard to survival in NSCLC patients with surgery whereas the social functioning scale and symptom scales of fatigue, appetite loss, and financial difficulties of the QLQ C-30 were strong predictors in NSCLC patients without surgery. Using goodness of fit as the criterion, items of symptoms on the QLQ-C30 were the strongest predictors among them. None of QLQ-LC13 scales was significant predictors. After simultaneously taking all significant scales of the QLQ-C30 into account, nausea/vomiting for NSCLC patients with surgery and appetite loss for NSCLC patients without surgery were the most informative variables for an improved prediction of survival, besides the traditional clinical parameters. These findings suggest that prediction of the prognosis could be improved by considering these HRQOL scales along with the current standard clinical prognostic variables.

The nausea/vomiting scale in NSCLC patients with surgery and appetite loss in NSCLC patients without surgery seemed to capture all the prediction after simultaneously considering all the significant scales of the QLQ-C30 and QLQ-LC13. The emotional functioning and pain scales in NSCLC patients with surgery and social functioning, fatigue, and financial difficulties scales in NSCLC patients without surgery became insignificant, possibly because they were moderately correlated with significant scales, and they did not provide new information regarding survival after these 2 significant items were considered.

Several different aspects of pretreatment HRQOL have been associated with survival in advanced cancer [13-16], breast cancer [17-19], lung cancer [5-8], oesophageal cancer [20-22], hepatocellular cancer [23], and head and neck cancer [24]. Even though few studies have been carried out in lung cancer patients, our results are in agreement with those in studies of lung cancer patients. Herndon et al. in a study of 206 patients with advanced NSCLC using the QLQ-C30 and Duke-UNC Social Support Scale, found that in addition to the cancer type and dyspnoea, the pain scale was a prognostic factor of survival [5]. Similarly, Langendijk et al. in a study of 198 patients treated with external irradiation using the QLQ-C30, observed that global QOL was an independent prognostic factor, but the effect varied as a function of cancer clinical stage in a 3-year follow-up [6]. Montazeri et al. using the Nottingham Health Profile, the QLQ-C30 and the QLQ-LC13, reported that the global QLQ of the QLQ-C30, along with age and extent of disease, were the significant predictors of 3-month survival in 129 newly diagnosed lung cancer patients [7]. Recently, Dharma-Wardene et al. using the Functional Assessment of Cancer Therapy-General (FACT-G), found that the baseline FACT-G total score was a significant predictor of subsequent survival in 42 patients with advanced lung cancer planning to undergo palliative chemotherapy [8].

Previous studies indicated that pre-treatment HRQOL is a significant factor for survival time, and pre-treatment assessment of HRQOL could help physicians in their clinical decisions, as it directly relates to patients’ survival time. Our finding is that cross-sectional HRQOL of all treatment types is also a significant factor for survival outcome, and this has important implications: the assessment of HRQOL in lung cancer patients should be integrated into clinical practice and evaluated periodically to adequately monitor the outcome of any resulting treatment or regular follow-up visits. In addition, the independent prognostic value of the QLQ-C30 scales for these patients suggests that a better HRQOL score, reflecting fewer symptoms of appetite loss would result in better overall survival in NSCLC patients. In our opinion, one of the therapeutic goals of HRQOL is to facilitate communication between patients and doctors, and help doctors provide care based on patients’ symptoms with the aim of improving overall survival by controlling the impact of disease and preserving or improving HRQOL.

The major strengths of our study are that all of the HRQOL data were available at baseline, with a less than 5% missing rate. The clinical characteristics of these patients were similar to those of the entire population of our clinical settings. A high standard of follow-up was applied, resulting in a low rate of loss to follow-up, a large number of events, and adequate overall statistical power. Furthermore, HRQOL was assessed using multidimensional QOL, which would be more informative than global QOL.

Our results should be interpreted with caution because of 5 limitations. One limitation is that some of deaths could not be confirmed through follow-up phone calls and had to be confirmed through linking with the National Mortality database. The death status of some patients with a missing identification number could not be confirmed. With only a few cases being lost to follow-up and with few missing identification numbers, the error should be small. Another potential drawback is that the analyses only considered baseline HRQOL; the relationship between changes in HRQOL measures and survival could not be examined. Future studies should focus on how the changes in HRQOL measures relate to survival due to any causes. Third, the study lacked information regarding pulmonary function, comorbidity, genetic factors and the physical environment of these patients; hence, we cannot rule out the possibility that our findings may be confounded due to these unmeasured variables. Fourth, due to limited number of sample size, we could not examine the relationship between HRQOL and survival in small cell lung cancer patients. Last, this study did not establish a causal relationship between HRQOL and survival, since an unidentified factor may have been involved. HRQOL may reflect a patient’s status of disease, which has a direct effect on a patient’s survival.

## Conclusion

In conclusion, our findings from this study support the use of HRQOL assessment in routine clinical practice in a NSCLC patient population. Although the data were prospectively collected, the findings are limited by the cross-sectional assessment of HRQOL. Further studies incorporating longitudinal HRQOL assessment together with clinical prognostic factors may provide additional information that may aid the patient and clinician in decision-making in NSCLC.

## Competing interests

The authors declare that they have no competing interests.

## Authors’ contributions

TCL and CCL contributed equally to the design of the study and the direction of its implementation, including supervision of the field activities, quality assurance and control. CHT, KSL, CYC and TCH supervised the field activities. CCL and YDL helped conduct the literature review and prepare the Methods and the Discussion sections of the text. TCL, CIL SYY and CCL designed the study’s analytic strategy and conducted the data analysis. All authors read and approved the final manuscript.

## Pre-publication history

The pre-publication history for this paper can be accessed here:

http://www.biomedcentral.com/1471-2458/12/790/prepub
